# Sulfate-Source-Dependent Anodic Discharge and Apparent Corrosion Response of Al Alloy Electrodes in Alkaline Electrolytes

**DOI:** 10.3390/ijms27156950

**Published:** 2026-08-02

**Authors:** Soon-Ki Jeong, Sohyun Kim, Yeonwoo Chung, Sangyup Lee, Seunga Yang

**Affiliations:** 1 Department of Future Convergence Technology, Graduate School, Soonchunhyang University, Soonchunhyang-ro 22-gil, Sinchang-myeon, Asan-si 31538, Chungcheongnam-do, Republic of Korea; chung@elech.kuic.kyoto-u.ac.jp (Y.C.); 20237450@sch.ac.kr (S.L.); tmddk123@sch.ac.kr (S.Y.); 2 Department of Energy Engineering, Soonchunhyang University, Soonchunhyang-ro 22-gil, Sinchang-myeon, Asan-si 31538, Chungcheongnam-do, Republic of Korea; 20181594@sch.ac.kr; 3 Advanced Energy Research Center, Soonchunhyang University, Soonchunhyang-ro 22-gil, Sinchang-myeon, Asan-si 31538, Chungcheongnam-do, Republic of Korea

**Keywords:** aluminum–air batteries, aluminum alloy anode, sulfate-source formulation, mass-loss-normalized anodic charge, apparent corrosion response, electrochemical impedance spectroscopy

## Abstract

Anodic corrosion of Al alloy electrodes limits their use as anodes in alkaline aluminum–air batteries. Na_2_SO_4_- and Al_2_(SO_4_)_3_-containing formulations were compared at matched nominal sulfate-group inputs but different formulation-derived Na and Al inputs. Aqueous 2 M NaOH was mixed with 0.3 M Na_2_SO_4_ or 0.1 M Al_2_(SO_4_)_3_ solution at NaOH:additive-solution volume ratios of 7:3, 5:5, and 3:7, and Al–Mg–Sn–Gd–P alloy electrodes were evaluated in a three-electrode configuration. All galvanostatic tests passed the same external charge of 160 mAh. Mass-loss-normalized anodic charge (Q_Δm_) is reported as an operational metric normalized by the net post-discharge electrode mass decrease, not as conventional battery capacity or efficiency. The Al_2_(SO_4_)_3_-containing formulations showed higher Q_Δm_ values; at the 3:7 ratio, Q_Δm_ increased from 917 to 1894 mAh g^−1^. Potentiodynamic polarization yielded lower polarization-derived apparent corrosion current densities and higher polarization resistances, while potentiostatic electrochemical impedance spectroscopy showed larger interfacial impedance responses in the Al_2_(SO_4_)_3_-containing series. Scanning electron microscopy showed electrolyte-dependent post-discharge morphologies. These results establish sulfate-source-dependent differences in anodic, polarization, impedance, and morphological responses under matched nominal sulfate-group input without isolating the effect of a single Na- or Al-containing species, establishing the presence or chemical identity of a distinct interfacial film, or demonstrating improved full-cell performance.

## 1. Introduction

Aqueous metal–air batteries have attracted attention as primary energy-conversion systems because they use oxygen from air as the cathodic active material and metal oxidation as the anodic reaction [1,2]. Among various metal–air systems, aluminum–air batteries (AABs) are particularly attractive owing to the high theoretical energy density of aluminum, its low cost, natural abundance, and the recyclability of Al-containing reaction products [3,4,5]. These characteristics make AABs relevant to high-capacity primary power sources, including emergency power supplies and mechanically rechargeable energy systems [3,4]. However, the practical performance of AABs remains below theoretical expectations because the Al anode undergoes surface passivation and parasitic corrosion in aqueous electrolytes [3,6].

In alkaline media, aluminum can be electrochemically oxidized to soluble aluminate species according to the anodic reaction [3,6]:

Al + 4OH^−^ → Al(OH)_4_^−^ + 3e^−^ (1)

This reaction enables anodic discharge by dissolving the surface oxide layer and activating the Al electrode. However, the same strongly alkaline environment promotes self-corrosion of Al through reactions involving water and hydroxide ions, which consume active Al without contributing to useful discharge [3,6]. The corrosion reaction is commonly accompanied by hydrogen evolution, leading to reduced anodic utilization, lower discharge capacity, and lower practical energy output [6,7]. Therefore, controlling the Al/electrolyte interface in alkaline media is a key requirement for improving the electrochemical utilization of Al alloy anodes.

Several strategies have been proposed to mitigate these limitations. Alloying Al with elements such as Mg, Sn, Ga, In, or rare-earth elements can modify the native oxide film and improve anodic activation [3,4]. However, alloy design alone cannot fully suppress electrolyte-driven corrosion because the corrosion process is strongly affected by the local chemical environment at the Al/electrolyte interface. Consequently, electrolyte engineering has become an important approach for controlling Al anode corrosion. Water-in-salt electrolytes, hydrogen-bonding additives, organic inhibitors, and interfacial film-forming additives have been reported to reduce the activity of free water molecules, modify the solvation environment, or suppress direct contact between aggressive species and the Al surface [1,7,8]. Our previous study further showed that alkali metal cations can influence anodic corrosion by altering water activity and interfacial resistance in alkaline AAB electrolytes [9]. Together, these studies indicate that electrolyte composition can regulate both molecular interactions in solution and interfacial electrochemical processes at the Al anode.

Sulfate-containing additives have been reported to influence Al dissolution in alkaline media. Prior studies have discussed competitive adsorption between sulfate and hydroxide ions and possible sulfate-containing interfacial species [7,10,11]. These literature-based pathways indicate that sulfate precursor identity can alter solution composition and interfacial response; however, they do not establish that a chemically defined sulfate-containing film forms under the present conditions. Moreover, the electrochemical response may depend on precursor stoichiometry, acid–base reactions, and the accompanying Na and Al inputs, not solely on the nominal sulfate-group input.

Aluminum sulfate, Al_2_(SO_4_)_3_, and sodium sulfate, Na_2_SO_4_, provide a useful pair of sulfate precursors for examining formulation-dependent effects. At each matched volume ratio, the formulations were designed to contain the same nominal sulfate-group input and the same nominal NaOH input, but they differed in their formula-based total Na and Al inputs. Under strongly alkaline conditions, Al_2_(SO_4_)_3_ should not be interpreted as producing a known concentration of freely solvated Al^3+^ because hydrolysis, aluminate formation, complexation, and acid–base neutralization can occur [12,13]. The comparison therefore evaluates sulfate-source formulation as a coupled electrolyte variable rather than isolating a single cation effect or assuming a chemically resolved equilibrium speciation.

In this study, Na_2_SO_4_- and Al_2_(SO_4_)_3_-containing formulations were compared at stoichiometrically matched nominal sulfate-group inputs to examine sulfate-source-dependent differences in the anodic response of Al–Mg–Sn–Gd–P alloy electrodes in alkaline electrolytes. Galvanostatic discharge, potentiodynamic polarization, potentiostatic electrochemical impedance spectroscopy (PEIS), and scanning electron microscopy/energy-dispersive X-ray spectroscopy (SEM/EDS) were used to evaluate the mass-loss-normalized anodic, polarization-derived apparent corrosion, interfacial impedance, and post-discharge morphological responses. Bulk apparent pH readings were retained only as descriptive electrolyte data and were not interpreted as measurements of local interfacial pH or thermodynamic OH^−^ activity. The electrolyte formulations and fixed-charge galvanostatic protocol are detailed in Section 3.2 and Section 3.3, respectively. This design treats sulfate source as a formulation-level electrolyte variable while distinguishing direct observations from unresolved mechanistic questions.

## 2. Results and Discussion

### 2.1. Galvanostatic Anodic Discharge and Mass-Loss-Normalized Anodic Charge in Sulfate-Containing Alkaline Electrolytes

Galvanostatic anodic discharge was used to compare Al–Mg–Sn–Gd–P alloy electrodes in matched Na_2_SO_4_- and Al_2_(SO_4_)_3_-containing formulations. The three-electrode configuration and the initial morphology of the polished Al alloy surface are shown in Figure S1a,b, respectively. Each test was conducted at 10 mA cm^−2^ over an exposed area of 3.2 cm^2^ for 5 h, corresponding to a total current of 32 mA and the same external charge of 160 mAh under every electrolyte condition. The matched formulations had the same nominal sulfate-group input at each volume ratio but different formulation-derived total Na and Al inputs, as detailed in Section 3.2.

**Figure 1:**
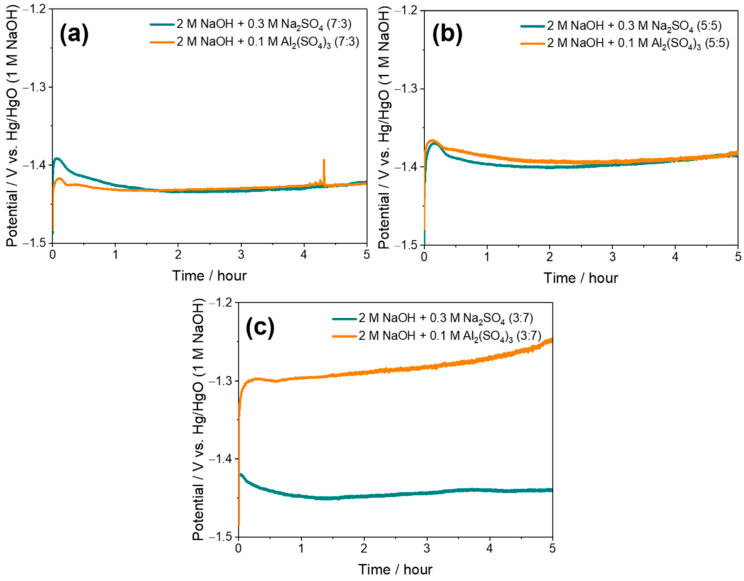
Galvanostatic anodic discharge curves of Al alloy electrodes recorded at 10 mA cm^−2^ in sulfate-containing alkaline electrolytes. The panels compare Na_2_SO_4_- and Al_2_(SO_4_)_3_-containing formulations at (**a**) 7:3, (**b**) 5:5, and (**c**) 3:7 volume ratios. Each test was conducted over 3.2 cm^2^ for 5 h and passed the same external charge of 160 mAh. Q_Δm_ denotes the operational mass-loss-normalized anodic charge summarized in Table 1; M denotes mol dm^−3^.

Figure 1a–c show the 5 h galvanostatic anodic discharge curves for the matched 7:3, 5:5, and 3:7 formulation pairs, respectively, and Table 1 summarizes the corresponding parameters. The mass-loss-normalized anodic charge, Q_Δm_, was 507 and 575 mAh g^−1^ for N30 and A30, 623 and 902 mAh g^−1^ for N50 and A50, and 917 and 1894 mAh g^−1^ for N70 and A70, respectively. The average anode potential remained between −1.44 and −1.39 V vs. Hg/HgO (1 M NaOH) in the Na_2_SO_4_-containing formulations and shifted from −1.43 to −1.28 V vs. Hg/HgO (1 M NaOH) with increasing Al_2_(SO_4_)_3_-solution fraction. Because the same external charge was passed in every test, differences in Q_Δm_ arise from the retained net-mass normalization rather than differences in total charge.

The higher Q_Δm_ values in the Al_2_(SO_4_)_3_-containing series support an operational formulation-to-formulation comparison under an identical external charge. Q_Δm_ is not interpreted as conventional battery capacity, current efficiency, or Faradaic efficiency because the net mass change does not partition useful electrochemical Al oxidation from hydrogen-evolution-associated corrosion, dissolution of alloy constituents, or retention and removal of surface products. In addition, the matched pairs differed in formulation-derived Na and Al inputs despite having the same nominal sulfate-group input. The discharge data therefore establish a sulfate-source-dependent mass-loss-normalized anodic response without isolating a single ionic or mechanistic contribution.

### 2.2. Potentiodynamic Polarization-Derived Apparent Corrosion Response

**Figure 2:**
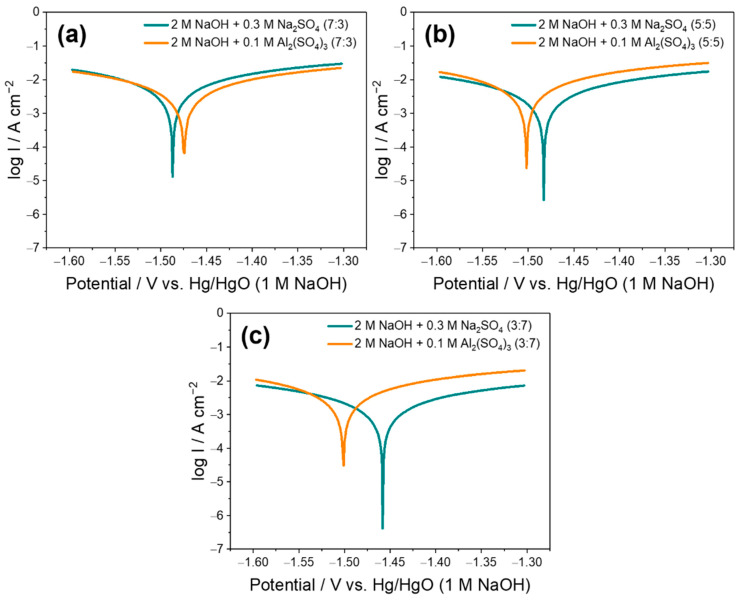
Potentiodynamic polarization curves of Al alloy electrodes in sulfate-containing alkaline electrolytes. The panels compare Na_2_SO_4_- and Al_2_(SO_4_)_3_-containing formulations at (**a**) 7:3, (**b**) 5:5, and (**c**) 3:7 volume ratios. Potentials are reported versus Hg/HgO (1 M NaOH). I denotes the absolute current density expressed in A cm^−2^ and the polarization-derived apparent corrosion parameters are summarized in Table 2. M denotes mol dm^−3^.

Potentiodynamic polarization measurements were conducted to compare the polarization-derived apparent corrosion response of the Al alloy electrodes. Figure 2a–c show the curves for the matched 7:3, 5:5, and 3:7 formulation pairs, and Table 2 summarizes the corresponding parameters [14].

For all matched volume ratios, the Al_2_(SO_4_)_3_-containing formulations showed lower polarization-derived apparent corrosion current densities (I_corr_) and higher polarization resistances (R_p_) than the corresponding Na_2_SO_4_-containing formulations. Replacing Na_2_SO_4_ with Al_2_(SO_4_)_3_ decreased I_corr_ from 20.52 to 15.63 mA cm^−2^ at 7:3, from 18.97 to 12.02 mA cm^−2^ at 5:5, and from 11.84 to 6.479 mA cm^−2^ at 3:7. In parallel, R_p_ increased from 2.545 to 3.151 Ω cm^2^, from 2.634 to 4.270 Ω cm^2^, and from 4.087 to 8.643 Ω cm^2^, respectively. At the 3:7 ratio, the corrosion potential (E_corr_) shifted from −1.498 to −1.455 V vs. Hg/HgO (1 M NaOH).

The lower I_corr_ and higher R_p_ values establish a lower polarization-derived apparent corrosion response for the Al_2_(SO_4_)_3_-containing formulations under the applied analysis conditions. These parameters reflect the coupled anodic and cathodic polarization response but do not directly quantify hydrogen evolution, mass-loss corrosion rate, or useful-current efficiency. Their co-variation with Q_Δm_ is therefore treated as a parallel electrochemical observation rather than proof of a common causal mechanism.

### 2.3. Electrochemical Impedance Response of the Al Alloy/Electrolyte Interface

**Figure 3:**
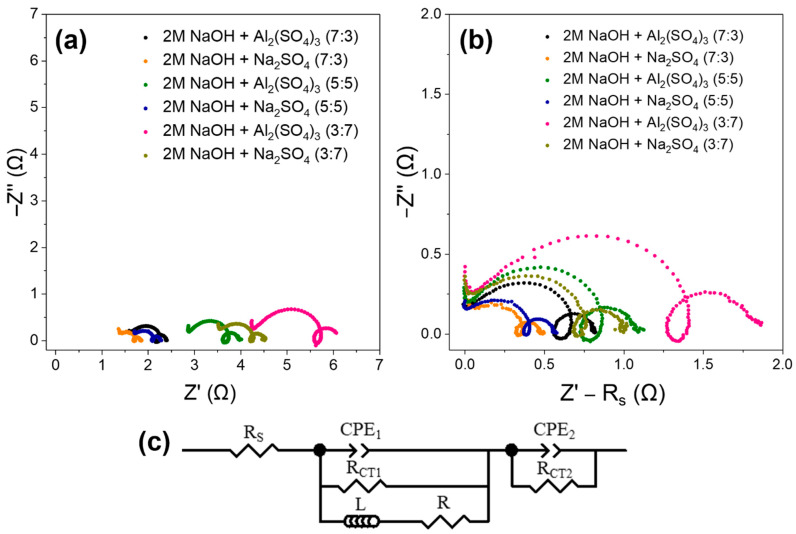
Electrochemical impedance response of Al alloy electrodes in sulfate-containing alkaline electrolytes. (**a**) Raw Nyquist spectra; (**b**) corresponding R_s_-subtracted spectra, with R_s_ determined from the high-frequency real-axis intercept of each raw spectrum and Z′_corr_ = Z′ − R_s_; and (**c**) equivalent-circuit representation used only to guide qualitative interpretation of the impedance features. Panel (**b**) compares arc shapes after removal of the ohmic real-axis offset and does not show the raw absolute-impedance positions. Z′ and Z″ denote the real and imaginary components of impedance, respectively; R_s_ denotes solution resistance; R_CT1_ and R_CT2_ denote the resistances associated with the high- and low-frequency charge-transfer branches, respectively; CPE_1_ and CPE_2_ denote constant-phase elements; L denotes inductance; and R denotes the resistance associated with the inductive branch. M denotes mol dm^−3^.

PEIS was conducted to compare the interfacial electrochemical response of the Al alloy electrodes. Figure 3a presents the raw Nyquist spectra, whereas Figure 3b presents the corresponding R_s_-subtracted spectra. For each spectrum, R_s_ was determined from the high-frequency real-axis intercept and the real component was corrected according to Z′_corr_ = Z′ − R_s_, while Z″ was unchanged. Figure 3c presents an equivalent-circuit representation used to guide qualitative interpretation.

The Al_2_(SO_4_)_3_-containing formulations showed larger observed impedance arcs than the matched Na_2_SO_4_-containing formulations. The present PEIS data therefore show a sulfate-source-dependent difference in interfacial impedance response. Because PEIS is not chemical-state-resolved, the larger response is not attributed to a distinct film, a specific sulfate-containing species, or suppression of a particular corrosion pathway. Charge-transfer and adsorbed-intermediate processes are treated only as literature-consistent possibilities [15,16], and the analysis is restricted to qualitative comparison of the measured spectra. Quantitative fitting parameters were not used as primary mechanistic evidence. The directly supported finding is the formulation-dependent difference in interfacial impedance response, not the chemical identity of its origin.

### 2.4. Post-Discharge Surface Morphology

**Figure 4:**
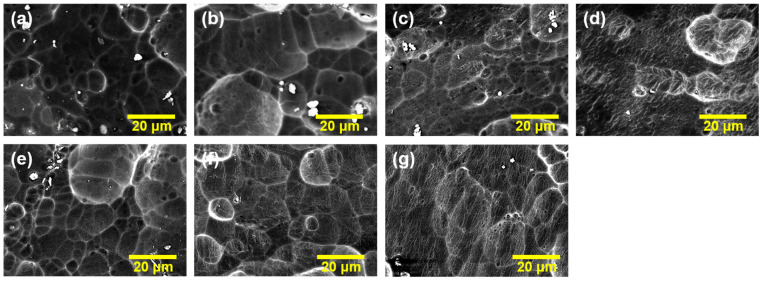
Representative post-discharge scanning electron microscopy (SEM) images of Al alloy electrodes after 5 h of anodic discharge in (**a**) 2 M NaOH, (**b**) 2 M NaOH + 0.1 M Al_2_(SO_4_)_3_ (7:3 vol.), (**c**) 2 M NaOH + 0.1 M Al_2_(SO_4_)_3_ (5:5 vol.), (**d**) 2 M NaOH + 0.1 M Al_2_(SO_4_)_3_ (3:7 vol.), (**e**) 2 M NaOH + 0.3 M Na_2_SO_4_ (7:3 vol.), (**f**) 2 M NaOH + 0.3 M Na_2_SO_4_ (5:5 vol.), and (**g**) 2 M NaOH + 0.3 M Na_2_SO_4_ (3:7 vol.). M denotes mol dm^−3^, and vol. denotes the NaOH:additive-solution volume ratio. The images are used only to compare representative electrolyte-dependent morphologies; chemical-state assignments require surface-sensitive analysis.

The post-discharge morphology of the Al alloy electrodes was examined by SEM to evaluate electrolyte-dependent surface evolution. Figure 4 compares the morphologies obtained after discharge in 2 M NaOH and in the sulfate-containing alkaline electrolytes, enabling sulfate-source effects to be assessed across the matched electrolyte series. The polished pristine Al alloy exhibited a relatively smooth morphology without large cracks or corrosion pits, as shown in Figure S1b. After discharge in 2 M NaOH, the surface exhibited pits and cracks, consistent with localized Al dissolution in strongly alkaline media [3,6].

The SEM observations showed formulation-dependent differences in post-discharge surface morphology, but the present surface SEM data do not establish a discrete layer or its chemical state, bonding, phase, thickness, or continuity. Figure S2 provides qualitative SEM/EDS elemental-distribution data for selected pristine and 1 h-discharge samples; these samples do not constitute a complete matched Na_2_SO_4_/Al_2_(SO_4_)_3_ comparison and represent a different electrochemical history from the 5 h SEM samples in Figure 4. Accordingly, the EDS data are treated only as qualitative elemental-distribution evidence [17].

### 2.5. Bulk Apparent pH Readings as Descriptive Electrolyte Parameters

The bulk apparent pH readings of the base solution, additive stock solutions, and final mixed electrolytes are summarized in Table 3. The 0.3 M Na_2_SO_4_ stock solution gave a reading of 6.79, whereas the 0.1 M Al_2_(SO_4_)_3_ solution gave a reading of 3.15. After mixing with 2 M NaOH, all final electrolytes remained strongly alkaline. The Na_2_SO_4_-containing formulations gave readings of 13.63, 13.59, and 13.49 at 7:3, 5:5, and 3:7, respectively, whereas the corresponding Al_2_(SO_4_)_3_-containing formulations gave readings of 13.59, 13.49, and 13.20.

The readings were obtained in concentrated alkaline matrices with different ionic compositions and were retained only to document one measured property of the tested formulations. They were not used to establish a causal relationship with Q_Δm_, the polarization-derived parameters, or the PEIS response. No pH-matched or statistical relationships were inferred from these readings.

The local interfacial pH during polarization and galvanostatic discharge was not measured and may differ from the bulk value because of reaction fluxes and mass transport. Because the revised interpretation does not rely on a causal local-pH mechanism, no near-surface simulation was introduced. Spatially resolved pH measurements and experimentally validated reaction–transport modeling would be appropriate for a separate mechanistic study.

### 2.6. Integrated Phenomenological Interpretation and Limitations

Across the matched formulations, the Al_2_(SO_4_)_3_-containing series generally exhibited higher Q_Δm_, lower polarization-derived apparent I_corr_, higher R_p_, larger interfacial impedance responses, and distinct post-discharge morphologies relative to the corresponding Na_2_SO_4_-containing series. These concurrent differences establish sulfate-source-dependent co-variation under the tested conditions but do not identify a unique causal sequence.

The matched formulations contained the same nominal sulfate-group inputs but different formulation-derived Na and Al inputs, and the contributions of these coupled variables were not independently separated. In addition, the PEIS spectra were obtained after 1 h immersion at OCP, whereas the main SEM images were obtained after 5 h of galvanostatic discharge. The two measurements therefore represent different electrochemical histories and should not be interpreted as observations of the same chemically defined interface.

The present measurements did not establish the presence of a distinct interfacial film or identify a sulfate bonding state, an Al-containing sulfate phase, or a layer thickness. The results are therefore interpreted as formulation-dependent electrochemical and morphological responses without assigning sulfate adsorption, Al-containing hydroxo/sulfate species, or film formation as the mechanism.

## 3. Materials and Methods

### 3.1. Materials and Electrode Preparation

The Al–Mg–Sn–Gd–P alloy specimens were supplied by ALUS Co., Ltd. (Cheonan, Republic of Korea). The nominal composition of the alloy was Al–Mg–Sn–Gd–P, with respective weight percentages of 99.312, 0.56, 0.01, 0.024, and 0.004 wt%, identical to that used in our previous study [9]. The alloy specimens were cut into rectangular electrodes with dimensions of 1.0 cm × 1.5 cm × 0.4 cm. Before electrochemical measurements, the native oxide layer on the electrode surface was removed by mechanical polishing using a polishing machine (RB209 MINIPOL; R&B Inc., Daejeon, Republic of Korea) and a polishing cloth (MPC-8A-Toplap; R&B Inc., Daejeon, Republic of Korea). The polished electrodes were ultrasonically cleaned in ethanol (94.5%; Daejung, Siheung, Republic of Korea) for 30 min and then dried in an oven at 60 °C for 1 h. Sodium hydroxide (NaOH, ACS reagent, ≥97.0%, pellets; Sigma-Aldrich, St. Louis, MO, USA), sodium sulfate (Na_2_SO_4_, ACS reagent, ≥99.0%, anhydrous, granular; Sigma-Aldrich, St. Louis, MO, USA), and aluminum sulfate octadecahydrate (Al_2_(SO_4_)_3_·18H_2_O, 98%; Sigma-Aldrich, St. Louis, MO, USA) were used as received. Distilled water (HPLC grade; Burdick & Jackson, Muskegon, MI, USA) was used to prepare all aqueous solutions.

### 3.2. Preparation of Electrolytes

The base solution was 2 mol dm^−3^ NaOH. Two sulfate-containing additive stock solutions were prepared separately: 0.3 mol dm^−3^ Na_2_SO_4_ and 0.1 mol dm^−3^ Al_2_(SO_4_)_3_. The Al_2_(SO_4_)_3_ solution was prepared from Al_2_(SO_4_)_3_·18H_2_O, and the stated molarity denotes the nominal concentration of Al_2_(SO_4_)_3_ formula units. The two stock solutions therefore contained the same nominal sulfate-group concentration of 0.30 mol dm^−3^ before mixing. The base solution was mixed with each stock solution at NaOH:additive-solution volume ratios of 7:3, 5:5, and 3:7. The resulting formulations were denoted N30, N50, and N70 for Na_2_SO_4_ and A30, A50, and A70 for Al_2_(SO_4_)_3_; the numbers indicate the additive-solution volume percentage. At each matched ratio, the N and A formulations had the same nominal NaOH and sulfate-group inputs but different formula-based total Na and Al inputs, as summarized in Table 4. These values are formulation-derived nominal inputs and do not represent measured total-element concentrations, free-ion concentrations or activities, residual OH^−^ concentration, ionic strength, or equilibrium speciation after mixing, hydrolysis, complexation, aluminate formation, and acid–base neutralization.

### 3.3. Galvanostatic Anodic Discharge Measurements

Galvanostatic anodic discharge measurements were conducted using a battery-test system (WBCS 3000; WonATech, Seoul, Republic of Korea) in a beaker-type three-electrode cell. The polished Al alloy electrode was the working electrode, a Pt mesh electrode (002250; ALS Co., Ltd., Tokyo, Japan) was the counter electrode, and an Hg/HgO/OH^−^ reference electrode filled with 1 M NaOH (RE-61AP, 013694; ALS Co., Ltd., Tokyo, Japan) was the reference electrode. The cell configuration is shown in Figure S1a. The exposed area was 3.2 cm^2^. Discharge was conducted at 10 mA cm^−2^ for 5 h, corresponding to 32 mA and 160 mAh.

Before discharge, the pre-discharge open-circuit potential was measured. During discharge, the anode potential was recorded versus Hg/HgO (1 M NaOH). After discharge, the Al alloy electrodes were rinsed with distilled water, dried at 60 °C for 1 h, and weighed. The net post-discharge mass decrease, Δm, was obtained from the difference between the initial and final electrode masses. The mass-loss-normalized anodic charge was calculated using Equation (2):

Q_Δm_ = (j × A × t)/Δm (2)

where Q_Δm_ is the mass-loss-normalized anodic charge (mAh g^−1^), j is the discharge current density (mA cm^−2^), A is the exposed area (cm^2^), t is the discharge time (h), and Δm is the net post-discharge electrode mass decrease (g). Q_Δm_ is retained as an operational metric normalized by the net post-discharge electrode mass decrease and is not interpreted as conventional battery capacity, current/Faradaic efficiency, direct useful-Al-oxidation fraction, or corrosion loss.

### 3.4. Potentiodynamic Polarization Measurements

Potentiodynamic polarization measurements were performed using an electrochemical workstation (WBCS 3000; WonATech, Seoul, Republic of Korea) in the same beaker-type three-electrode cell used for galvanostatic discharge measurements.

The polished Al alloy electrode was the working electrode, a Pt mesh electrode was the counter electrode, and the Hg/HgO/OH^−^ reference electrode filled with 1 M NaOH described in Section 3.3 was the reference electrode. The exposed working-electrode area was 1.0 cm^2^.

The polarization scan was started at −1.50 V vs. Hg/HgO (1 M NaOH) and recorded at 1 mV s^−1^. E_corr_, I_corr_, and R_p_ were obtained by Tafel extrapolation using the same fitting procedure and potential-window criteria for all formulations.

The polarization resistance was calculated using the Stern–Geary relationship:

R_p_ = (β_a_ × β_c_)/[2.303 I_corr_(β_a_ + β_c_)] (3)

where β_a_ and β_c_ are the anodic and cathodic Tafel slopes, respectively, and I_corr_ is the polarization-derived apparent corrosion current density. The absolute Tafel-slope values were used in Equation (3). The resulting I_corr_ and R_p_ values were used only as polarization-derived apparent parameters for comparing the tested formulations.

### 3.5. Potentiostatic Electrochemical Impedance Spectroscopy

PEIS was conducted using an electrochemical workstation (ZIVE SP1; WonATech, Seoul, Republic of Korea) in the same three-electrode cell. Before each measurement, the Al alloy electrode was immersed under open-circuit conditions for 1 h at room temperature. PEIS was then performed with the DC bias set to the OCP measured after the 1 h immersion period using a sinusoidal potential perturbation of 5 mV over 100 kHz to 0.1 Hz. For every formulation, the absolute difference between the PEIS OCP and the corresponding pre-discharge OCP was 2 mV or lower.

The impedance spectra were presented as Nyquist plots and interpreted qualitatively using an equivalent-circuit representation. For Figure 3b, R_s_ was obtained from the high-frequency real-axis intercept of each raw spectrum and subtracted from Z′ according to Z′_corr_ = Z′ − R_s_, while Z″ was unchanged. This representation was only used to guide qualitative assignment of the impedance features; quantitative fitting parameters were not used as primary mechanistic evidence. The analysis was restricted to comparative interfacial impedance responses and did not determine film composition or chemical state.

### 3.6. Surface Morphology and Elemental Distribution Analysis

The surface morphology was examined by SEM (SIGMA 360; Carl Zeiss AG, Oberkochen, Baden-Württemberg, Germany). The polished pristine electrode and post-discharge electrodes were compared after the discharged samples had been rinsed with distilled water and dried at 60 °C. EDS (XFlash 7; Bruker Corporation, Billerica, MA, USA) mapping was performed on selected pristine, treated, and post-discharge samples. SEM/EDS was only used for representative morphology and qualitative elemental-distribution analysis and not to determine bonding, valence state, phase, film thickness, continuity, or the chemical origin of the impedance response.

### 3.7. Bulk Apparent pH Measurements

The bulk pH readings of the base solution, additive stock solutions, and final mixed electrolytes were obtained at room temperature using a pH meter (FP20; Mettler Toledo, Columbus, OH, USA). The same measurement protocol was applied to all solutions. Because the measurements were made in concentrated NaOH/sulfate matrices, the values are only reported as bulk apparent pH readings. They are not interpreted as thermodynamic OH^−^ activities, local interfacial pH values, or acid-neutralizing alkalinities and are not used as mechanistic evidence for the electrochemical conclusions.

## 4. Conclusions

This study compared Na_2_SO_4_- and Al_2_(SO_4_)_3_-containing alkaline formulations at matched nominal sulfate-group inputs but different formulation-derived Na and Al inputs. Under the identical external charge of 160 mAh, the Al_2_(SO_4_)_3_-containing series showed higher Q_Δm_ values, including an increase from 917 to 1894 mAh g^−1^ for the matched 3:7 pair. Potentiodynamic polarization further showed that the polarization-derived apparent I_corr_ decreased from 11.84 to 6.479 mA cm^−2^, while R_p_ increased from 4.087 to 8.643 Ω cm^2^ for the same pair. The Al_2_(SO_4_)_3_-containing series also showed larger interfacial impedance responses and distinct representative post-discharge morphologies. These results identify sulfate-source formulation as a practical electrolyte-design variable associated with distinct mass-loss-normalized anodic, polarization-derived apparent corrosion, impedance, and morphological responses under matched nominal sulfate-group input.

The observations do not isolate the contribution of a single Na- or Al-containing species, establish a protective film or sulfate bonding state, or define a common causal mechanism. Bulk apparent pH readings were measured as descriptive electrolyte properties but were not used to establish causality. Future work can address the unresolved origin of these responses through complete Al mass/charge-balance measurements, spatially resolved interfacial-pH measurements with validated reaction–transport modeling, and surface-sensitive chemical-state analysis. These analyses concern mechanistic questions beyond the comparative scope of the present study.

## Figures and Tables

**Table 1:** Galvanostatic anodic discharge parameters of Al alloy electrodes in sulfate-containing alkaline electrolytes.

Electrolyte Code	Electrolyte	Mass-Loss-Normalized Anodic Charge, Q_Δm_ (mAh g^−1^)	Pre-Discharge OCP [V vs. Hg/HgO (1 M NaOH)]	Average Anode Potential [V vs. Hg/HgO (1 M NaOH)]
N30	2 M NaOH + 0.3 M Na_2_SO_4_ (7:3 vol.)	507	−1.48	−1.44
A30	2 M NaOH + 0.1 M Al_2_(SO_4_)_3_ (7:3 vol.)	575	−1.48	−1.43
N50	2 M NaOH + 0.3 M Na_2_SO_4_ (5:5 vol.)	623	−1.48	−1.43
A50	2 M NaOH + 0.1 M Al_2_(SO_4_)_3_ (5:5 vol.)	902	−1.48	−1.39
N70	2 M NaOH + 0.3 M Na_2_SO_4_ (3:7 vol.)	917	−1.53	−1.39
A70	2 M NaOH + 0.1 M Al_2_(SO_4_)_3_ (3:7 vol.)	1894	−1.48	−1.28

Note: Each test was conducted at 10 mA cm^−2^ over 3.2 cm^2^ for 5 h, corresponding to 160 mAh. Q_Δm_ is an operational metric normalized by the net post-discharge electrode mass decrease. Because the net mass change can reflect useful anodic dissolution, hydrogen-evolution-associated corrosion, dissolution of alloy constituents, and retention or removal of surface products, Q_Δm_ does not resolve individual Faradaic or corrosion pathways and is not interpreted as conventional battery capacity or current/Faradaic efficiency. The open-circuit potential (OCP) values listed in Table 1 were measured before galvanostatic discharge and are distinct from the PEIS bias potentials measured after 1 h immersion.

**Table 2:** Potentiodynamic polarization parameters of Al alloy electrodes in sulfate-containing alkaline electrolytes.

Electrolyte Code	Electrolyte	E_corr_ [V vs. Hg/HgO (1 M NaOH)]	I_corr_ (mA cm^−2^)	R_p_ (Ω cm^2^)
N30	2 M NaOH + 0.3 M Na_2_SO_4_ (7:3 vol.)	−1.484	20.52	2.545
A30	2 M NaOH + 0.1 M Al_2_(SO_4_)_3_ (7:3 vol.)	−1.471	15.63	3.151
N50	2 M NaOH + 0.3 M Na_2_SO_4_ (5:5 vol.)	−1.499	18.97	2.634
A50	2 M NaOH + 0.1 M Al_2_(SO_4_)_3_ (5:5 vol.)	−1.480	12.02	4.270
N70	2 M NaOH + 0.3 M Na_2_SO_4_ (3:7 vol.)	−1.498	11.84	4.087
A70	2 M NaOH + 0.1 M Al_2_(SO_4_)_3_ (3:7 vol.)	−1.455	6.479	8.643

Note: E_corr_, corrosion potential; I_corr_, polarization-derived apparent corrosion current density; R_p_, polarization resistance. I_corr_ and R_p_ are polarization-derived apparent corrosion parameters; they are not direct hydrogen-evolution or mass-loss corrosion-rate measurements. N and A denote Na_2_SO_4_- and Al_2_(SO_4_)_3_-containing formulations, respectively, and the numbers indicate the volume percentage of the additive solution.

**Table 3:** Bulk apparent pH readings of the base solution, additive stock solutions, and final mixed electrolytes.

Solution Code	Electrolyte	Bulk Apparent pH Reading
Base	2 M NaOH	13.54
Na_2_SO_4_	0.3 M Na_2_SO_4_	6.79
Al_2_(SO_4_)_3_	0.1 M Al_2_(SO_4_)_3_	3.15
N30	2 M NaOH + 0.3 M Na_2_SO_4_	13.63
A30	2 M NaOH + 0.1 M Al_2_(SO_4_)_3_	13.59
N50	2 M NaOH + 0.3 M Na_2_SO_4_	13.59
A50	2 M NaOH + 0.1 M Al_2_(SO_4_)_3_	13.49
N70	2 M NaOH + 0.3 M Na_2_SO_4_	13.49
A70	2 M NaOH + 0.1 M Al_2_(SO_4_)_3_	13.20

Note: All final mixed electrolytes remained strongly alkaline. The values are reported only as bulk apparent pH readings obtained under the same measurement protocol; they are not interpreted as thermodynamic OH^−^ activities, local interfacial pH values, or acid-neutralizing alkalinities.

**Table 4:** Formulation-derived nominal composition of the final mixed electrolytes.

Solution Code	NaOH-to-Additive-Solution Volume Ratio	Nominal NaOH Introduced (mol dm^−3^)	Nominal Na_2_SO_4_ Introduced (mol dm^−3^)	Nominal Al_2_(SO_4_)_3_ Introduced (mol dm^−3^)	Formula-Based Total Na Introduced (mol dm^−3^)	Formula-Based Total Al Introduced (mol dm^−3^)	Total Nominal Sulfate Groups Introduced (mol dm^−3^)
N30	7:3	1.40	0.09	—	1.58	—	0.09
A30	7:3	1.40	—	0.03	1.40	0.06	0.09
N50	5:5	1.00	0.15	—	1.30	—	0.15
A50	5:5	1.00	—	0.05	1.00	0.10	0.15
N70	3:7	0.60	0.21	—	1.02	—	0.21
A70	3:7	0.60	—	0.07	0.60	0.14	0.21

Note: Values are formulation-derived nominal inputs calculated from precursor molarities and nominal volume fractions. Al_2_(SO_4_)_3_ values denote nominal formula-unit inputs calculated from the Al_2_(SO_4_)_3_·18H_2_O precursor. Formula-based Na and Al values denote stoichiometric elemental inputs, and sulfate values denote stoichiometric sulfate-group inputs. They do not represent measured total-element concentrations, freely solvated Na^+^ or Al^3+^ concentrations, uncomplexed sulfate concentration or activity, residual OH^−^ concentration or activity, ionic strength, or equilibrium species after mixing, hydrolysis, complexation, aluminate formation, and acid–base neutralization.

## Data Availability

The processed data presented in this study are available within the article and Supplementary Materials. The archived dataset contains the processed Q_Δm_ values reported in this study but not the individual unrounded initial and final mass entries needed to report the measured mass losses at their original precision. Accordingly, no separate mass-loss dataset was back-calculated from the rounded Q_Δm_ values or newly reported as measured data. Other retained data are available from the corresponding author upon reasonable request.

## Supplementary Materials

The following supporting information can be downloaded at: https://www.mdpi.com/article/10.3390/ijms27156950/s1.
