# Supplementary material for: Sulfate-Source-Dependent Anodic Discharge and Apparent Corrosion Response of Al Alloy Electrodes in Alkaline Electrolytes
Source: Int J Mol Sci. 2026 Aug 2;27(15):6950. doi: 10.3390/ijms27156950 (PMC13467159; doi:10.3390/ijms27156950)
Supplement: Supplementary file 1 [file ijms-27-06950-s001.zip › ijms-4424147-supplementary.pdf]

Supplementary Information figures

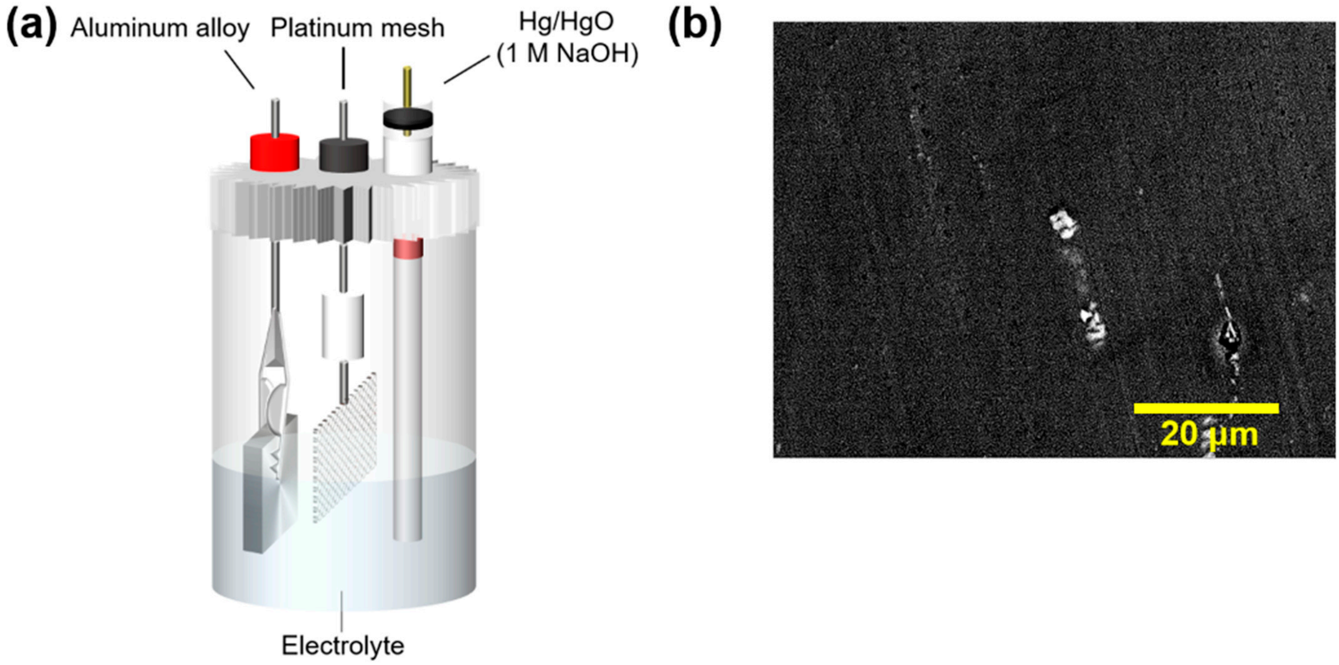

Figure S1. Experimental cell configuration and polished Al alloy surface. (a) Three-electrode cell used for electrochemical measurements, comprising the Al alloy working electrode, Pt mesh counter electrode, and Hg/HgO/OH^{-} reference electrode filled with 1 M NaOH. (b) scanning electron microscopy (SEM) image of the polished Al alloy surface before electrochemical testing. This figure supports the Materials and Methods section and is not used as a main-text result figure.

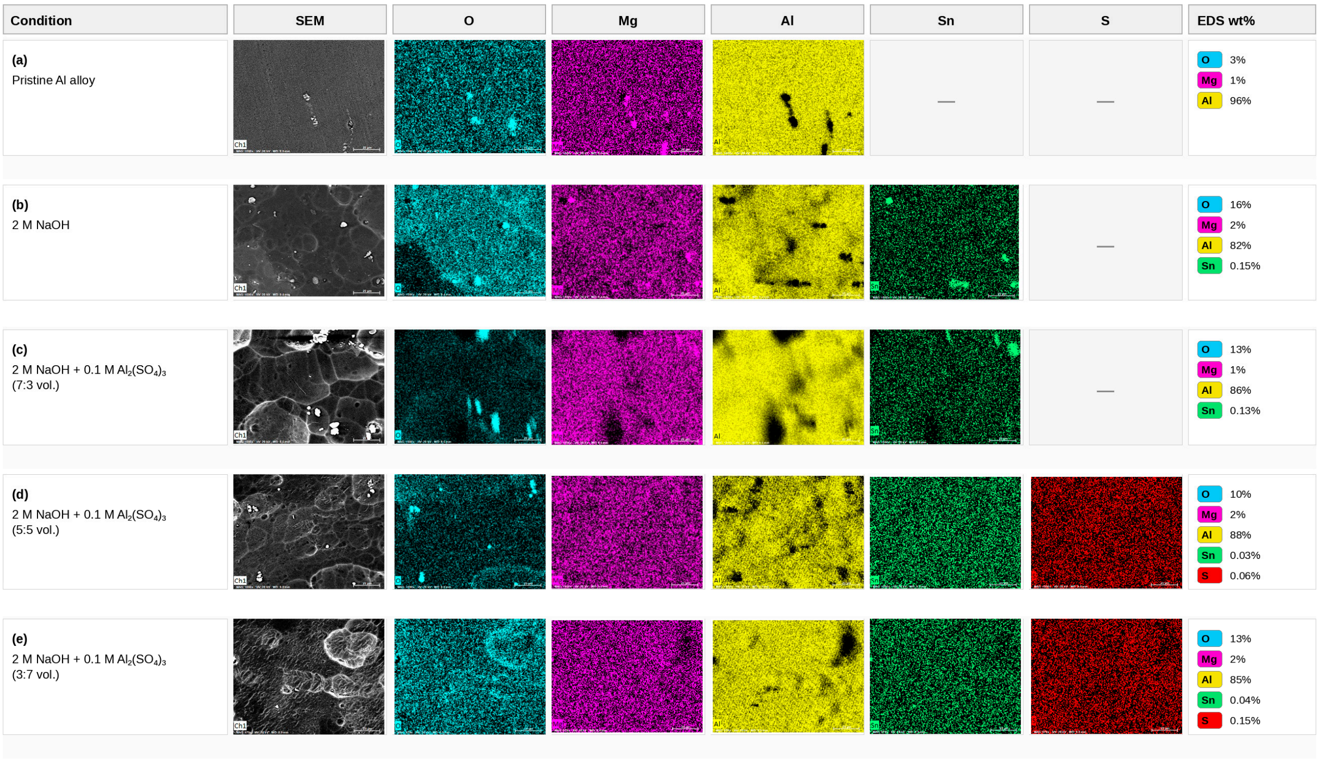

Figure S2. Scanning electron microscopy/energy-dispersive X-ray spectroscopy (SEM/EDS) mapping results of selected Al alloy electrodes before and after electrochemical treatment. Panel (a) shows the pristine Al alloy, whereas panels (b–e) show electrodes after 1 h anodic discharge in 2 M NaOH and $Al_2(SO_4)_3$-containing electrolytes at 7:3, 5:5, and 3:7 volume ratios, respectively. The EDS maps are provided only as supplementary elemental-distribution data. The selected panels do not constitute a complete matched Na_2SO_4/$Al_2(SO_4)_3$ comparison and represent a different electrochemical history from the 5 h SEM samples in Figure 4. Trace sulfur signals are reported only as elemental signals and are not used to assign sulfate bonding, film incorporation, surface chemical states, or the origin of the potentiostatic electrochemical impedance spectroscopy (PEIS) response. The instrument-reported EDS wt% values are shown only as semi-quantitative survey outputs and are not used to establish film composition, sulfate bonding, or surface chemical state.
